# An Innovative Strategy for Dual Inhibitor Design and Its Application in Dual Inhibition of Human Thymidylate Synthase and Dihydrofolate Reductase Enzymes

**DOI:** 10.1371/journal.pone.0060470

**Published:** 2013-04-05

**Authors:** Mahreen Arooj, Sugunadevi Sakkiah, Guang ping Cao, Keun Woo Lee

**Affiliations:** Division of Applied Life Science (BK21 Program), Systems and Synthetic Agrobiotech Center (SSAC), Plant Molecular Biology and Biotechnology Research Center (PMBBRC), Research Institute of Natural Science(RINS), Gyeongsang National University (GNU), Jinju, Republic of Korea; University of Bologna & Italian Institute of Technology, Italy

## Abstract

Due to the diligence of inherent redundancy and robustness in many biological networks and pathways, multitarget inhibitors present a new prospect in the pharmaceutical industry for treatment of complex diseases. Nevertheless, to design multitarget inhibitors is concurrently a great challenge for medicinal chemists. We have developed a novel computational approach by integrating the affinity predictions from structure-based virtual screening with dual ligand-based pharmacophore to discover potential dual inhibitors of human Thymidylate synthase (hTS) and human dihydrofolate reductase (hDHFR). These are the key enzymes in folate metabolic pathway that is necessary for the biosynthesis of RNA, DNA, and protein. Their inhibition has found clinical utility as antitumor, antimicrobial, and antiprotozoal agents. A druglike database was utilized to perform dual-target docking studies. Hits identified through docking experiments were mapped over a dual pharmacophore which was developed from experimentally known dual inhibitors of hTS and hDHFR. Pharmacophore mapping procedure helped us in eliminating the compounds which do not possess basic chemical features necessary for dual inhibition. Finally, three structurally diverse hit compounds that showed key interactions at both active sites, mapped well upon the dual pharmacophore, and exhibited lowest binding energies were regarded as possible dual inhibitors of hTS and hDHFR. Furthermore, optimization studies were performed for final dual hit compound and eight optimized dual hits demonstrating excellent binding features at target systems were also regarded as possible dual inhibitors of hTS and hDHFR. In general, the strategy used in the current study could be a promising computational approach and may be generally applicable to other dual target drug designs.

## Introduction

Drug design is the inventive process of finding new medications based on the knowledge of the biological target. The notion of ‘one molecule – one target – one disease’ has been a prevalent paradigm in pharmaceutical industry. The main idea of this approach is the identification of a single protein target whose inhibition leads to a successful treatment of the examined disease. The predominant assumption is that highly selective ligands would avoid unwanted side effects caused by binding to secondary non-therapeutic targets. Many successful drugs have been transpired from this procedure. However, the diligence of inherent redundancy and robustness in many biological networks and pathways depicts that inhibiting a single target might fall short of producing the desired therapeutic effect [1]–[3]. As simultaneous intervention of two or multiple targets relevant to a disease has shown improved therapeutic efficacy, there has been a move toward multiple target drugs [4]. Across the pharmaceutical industry, this strategy of multitarget drugs has become an active field and around 20 multitarget drugs have been approved or are in advanced development stages [5]. Multitarget therapeutic strategy can be used to inhibit two or more enzymes, act on an enzyme and a receptor, or affect an ion channel and a transporter. Multitarget therapeutic strategy can be accomplished by one of the following approaches: (i) acting upon different targets to create a combination effect (e.g., Bactrim, which acts on two targets in the folate biosynthesis pathway in bacteria), (ii) altering the ability of another to reach the target, and (iii) binding the different sites on the same target to create a combination effect [6]. Modulating multiple targets in the biological network simultaneously is renowned to be beneficial for treating a range of diseases, such as acquired immune deficiency syndrome (AIDS), atherosclerosis, cancer, and depression, and this recognition has escorted to a growing tendency to devise multiple-target drugs [7]–[9]. Several multicomponent drugs have been launched, such as (4 S,7 S,10a S)-5- oxo-4-{[(2 S)-3-phenyl-2-sulfanylpropanoyl]amino}-2,3,4,7,8,9,10,10a-octahydropyrido[6,1-] [1], [3]thiazepine-7-carboxylic acid (omapatrilat) (a dual angiotensin-converting enzyme and neutral endopeptidase inhibitor) and 5-((6-((2-fluorophenyl) methoxy)-2-naphthalenyl) methyl)-2,4-thiazolidinedione (netoglitazone) (a peroxisome proliferator-activated receptor (PPAR)-R and PPAR-γ agonist) [10]. Many multitarget drugs are in clinical use today, but the discovery process is serendipitous, and their modes of action are usually elucidated retrospectively. Although, there is an increasing interest in developing drugs that take effect on multiple targets but designing multitarget inhibitors with predefined biological profiles is concurrently a great challenge for medicinal chemists. A very few computer-aided multitarget methods have been introduced in designing multitarget drugs. For instance, early design strategies tried to link the pharmacophores of known inhibitors; however these methods often lead to high molecular weight and low ligand efficacy. Moreover, sequential docking has also been implemented in designing multitarget drugs [11]. However, this docking methodology is computationally expensive for large-scale database screening. Another computational methodology merging molecular docking with common pharmacophore mapping was also applied for design of multitarget drugs. But, this approach used a single conformation inhibitor-protein complex [12]. Thus, more effective computational methods for the identification and further optimization of multitarget drugs in a complex disease system are needed.

Drug discovery and development is a lengthy and costly process. Of special interest to us are the development and application of novel computational methods for lead generation and lead optimization in the drug discovery process. These computational methods are generally categorized as ligand-based and structure-based methods [13]. The uses of structure-based and ligand-based methods with rational drug discovery have been fairly separate approaches. Moderate resolution (at least 2.4 Å) three dimensional X-ray structures of drug targets are a prerequisite for structure-based drug design. These structures provide a starting point for rational drug design by defining the topography of the protein target that the ligand surface must complement. This information helps the synthetic chemist to optimize compounds so as to enhance their interactions with the protein, resulting in improved potency and selectivity). Indeed, there are now several drugs on the market that originated from this structure-based design approach. The most commonly cited are the human immunodeficiency virus (HIV) drugs, such as amprenavir (Agenerase) and nelfinavir (Viracept), which were developed using the crystal structure of HIV protease [14]. Moreover, designed according to information obtained from the crystallographic data of the viral NA complexed with its natural substrate sialic acid, zanamivir and oseltamivir present a big success in the history of rational, structure-based drug development [15]. Ligand-based computational methods are often employed when detailed structural information is not available for the target of interest, or the biological target is completely unknown as is the case in many phenotypic assay based discovery. When biological activities of multiple hits are known, a more sophisticated class of computational techniques known as pharmacophore identification methods is often employed to deduce the essential features required for the biological activity. The description of important chemical features in the shape of a pharmacophore model offers the advantage of a fast and reliable technique when the input data is of high quality (high affinity ligands, high resolution of the X-ray complex structure).

Keeping this in view, a new strategy amalgamating structure-based and ligand-based approaches to identify small molecules modulating multiple targets is presented in this research exertion. This strategy integrates the structure-based information about the key elements in protein-ligand binding with the dual ligand-based pharmacophore model derived from experimentally known dual inhibitors to design multitarget drugs. On one hand, the structure-based approach is able to present the interactions of a ligand to the target protein in a very specific way. On the other hand, the ligand-based pharmacophore modeling approach is not restricted to the bound conformation of the ligand in the crystalline complex and is also able to reveal the common demand of multiple ligands.

**Figure 1:**
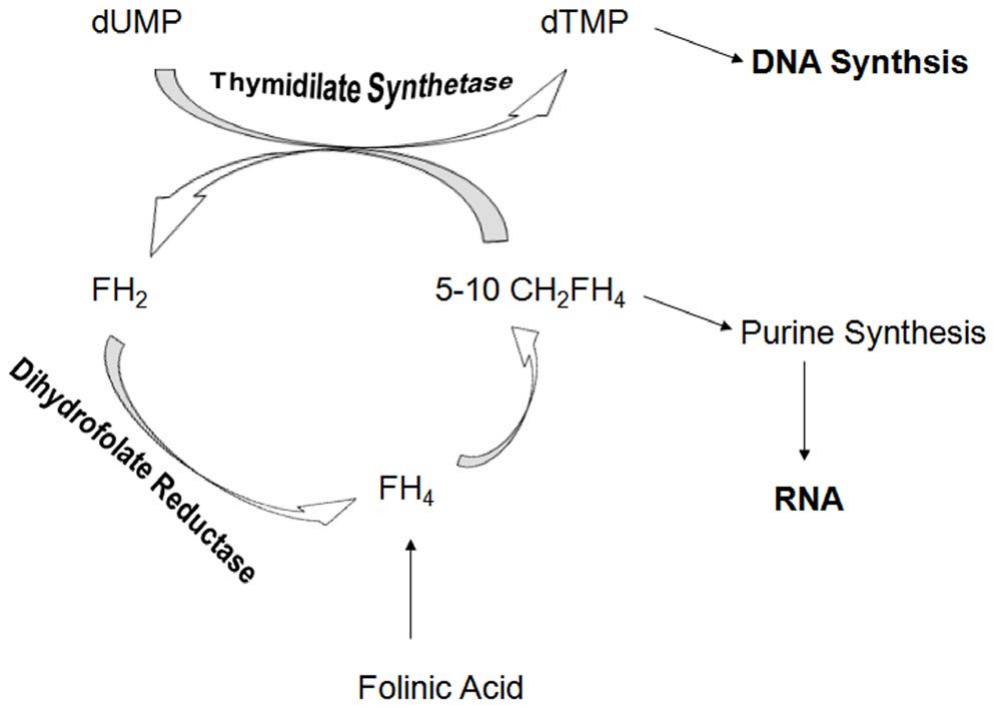
Conversion of deoxyuridine monophosphate (dUMP) into deoxythymidine monophosphate (dTMP), and dihydrofolate (FH2) to tetrahydrofolate (FH4) by TS and DHFR, respectively.

Thymidylate synthase (TS) (EC 2.1.1.45) and Dihydrofolate reductase (DHFR) (EC 1.5.1.3) are the key enzymes in folate metabolic pathway that is necessary for the biosynthesis of RNA, DNA, and protein [16], [17]. TS catalyzes the dUMP (2`-deoxyuridine-5`-monophosphate) methylation reaction involving a concerted transfer and the reduction of a single carbon with concomitant production of dTMP (thymidylate monophosphate) (one of the four building blocks of DNA) and dihydrofolate (DHF) (Figure 1). As, the reaction of TS is the sole intracellular source of de novo synthesized dTMP, therefore, inhibition of TS blocks DNA synthesis and prevents cellular proliferation [18]. For continuous production of dTMP in dividing cells, the oxidized 7,8-DHF must be converted back to 5,10-methylenetetrahydrofolate. Dihydrofolate reductase (DHFR) 5 catalyzes first of the two steps in this biotransformation in which NADPH acts as the source of the reductant. DHF is reduced to tetrahydrofolate (THF) via DHFR. DHFR is present in all cells and is necessary for the maintenance of intracellular folate pools in a biochemically active reduced state. Inhibition of DHFR results in depletion of intracellular reduced folates, which are necessary for one-carbon transfer reactions. One-carbon transfer reactions are important for the biosynthesis of thymidylate, purine nucleotides, methionine, serine, glycine, and many other compounds necessary for RNA, DNA, and protein synthesis [19]–[21]. The inhibition of DHFR depletes THF pools, causing genomic and proteomic instability and, ultimately, cell death. Consequently, targeting of both TS and DHFR for inactivation in tumor cells is an imperative approach in developing drugs for cancer chemotherapy. Several TS and DHFR inhibitors, as separate entities, have found clinical utility as antitumor agents [22]–[25]. One of the potent TS inhibitors, a dUMP analogue 5-fluoro-dUMP (FdUMP), is an active metabolite of a broadly used anticancer drug, 5-fluorouracil (5-FU). The DHFR inhibitor methotrexate (MTX) is one of the first chemotherapeutic agents discovered and is still a mainstay in single agent and combination cancer chemotherapy [26]. It has been reported that when a DHFR inhibitor is used in combination with a TS inhibitor, synergistic growth inhibition can occur against Lactobacillus casei, rat hepatoma cells, and human lymphoma cells [27], [28]. Inhibition of TS or of DHFR leads to “thymineless death” in the absence of salvage, and inhibition of these enzymes has found clinical utility as antitumor, antimicrobial, and antiprotozoal agents [17], [19], [20]. Dual inhibitors of TS and DHFR inhibitors could circumvent the pharmacokinetic disadvantages of two separate drugs. Therefore, to design single agents that could act as dual inhibitors of TS and DHFR is an important strategy in developing drugs for cancer chemotherapy.

## Materials and Methods

### Preparation of the Docking Library

Maybridge, a commercial chemical database containing 59 652 compounds, has been employed in this study for structure-based virtual screening procedure [29]. However, this database is found to have a number of nondruglike compounds. As, it is worthless to dock all the compounds of this database into the active site of protein target and then reject them in the later stage for their nondruglike properties, the compounds not satisfying druglike properties were excluded from the database prior to molecular docking (structure-based virtual screening). In order to accomplish this task, compounds in the database were subjected to various scrupulous druglike filters such as Lipinski’s rule of five and ADMET (absorption, distribution, metabolism, excretion, and toxicity) properties. *Prepare Ligands* and *ADMET Descriptors* protocols as available in Accelrys Discovery Studio v3.0 (DS), Accelrys, San Diego, USA program were used in this step.

### Preparation of Target Protein Systems for Docking

The necessity of providing a correct representation of the protein structure is a prerequisite for carrying out a successful docking study. Hence to ensure quality, the following aspects were considered. In this study, we have used the crystal structure of human DHFR (PDB ID: 1U72) determined at a resolution of 1.9 Å bound with inhibitor molecule Methotrexate (MTX) [30]. While, crystal structure with PDB ID of 1HVY co-crystallized with potent inhibitor TOMUDEX at a resolution of 1.9 Å was used for human TS enzyme [31]. The 3D coordinates of these enzymes were obtained from PDB and all the water molecules present in the structure of proteins were removed. Hydrogen atoms were added to the target protein structures using CHRAMM force field as available in DS followed by the minimization of added hydrogen atoms using *Smart Minimizer* protocol with a constraint on heavy atoms. The resulting target protein structures were used in molecular docking studies.

### Molecular Docking for hDHFR and hTS Inhibition

Virtual screening is emerging as a productive and cost-effective technology in rational drug design for the identification of novel lead compounds from large virtual database. In this study, a locally constructed druglike Maybridge database having a huge set of diverse compounds was used for docking into the active sites of hTS and hDHFR enzymes. Molecular docking studies were carried out using GOLD (Genetic Optimization for Ligand Docking) 5.1 program from Cambridge Crystallographic Data Center, UK. GOLD uses a genetic algorithm for docking ligands into protein binding sites to explore the full range of ligand conformational flexibility with partial flexibility of protein [32]. Molecular docking was performed to generate the bioactive binding poses of compounds in the active site of both enzymes. For both target systems, the active site was defined with a 10 Å radius around the ligand present in the corresponding crystal structure. Ten docking runs were performed per structure unless five of the 10 poses were within 1.5 Å RMSD of each other. All other parameters were kept at their default values. The GOLD fitness score is calculated from the contributions of hydrogen bond and van der Waals interactions between the protein and ligand, intramolecular hydrogen bonds and strains of the ligand. The interacting ability of a compound depends on the fitness score, greater the GOLD fitness score better the binding affinity. The protein–ligand interactions were examined by DS. Compounds which showed the key interactions with critical amino acids present in the active site of both target proteins and also exhibited higher GOLD fitness score were considered as possible dual hits and were selected for further evaluation.

### Generation and Validation of Common Feature Pharmacophore Model

**Figure 2:**
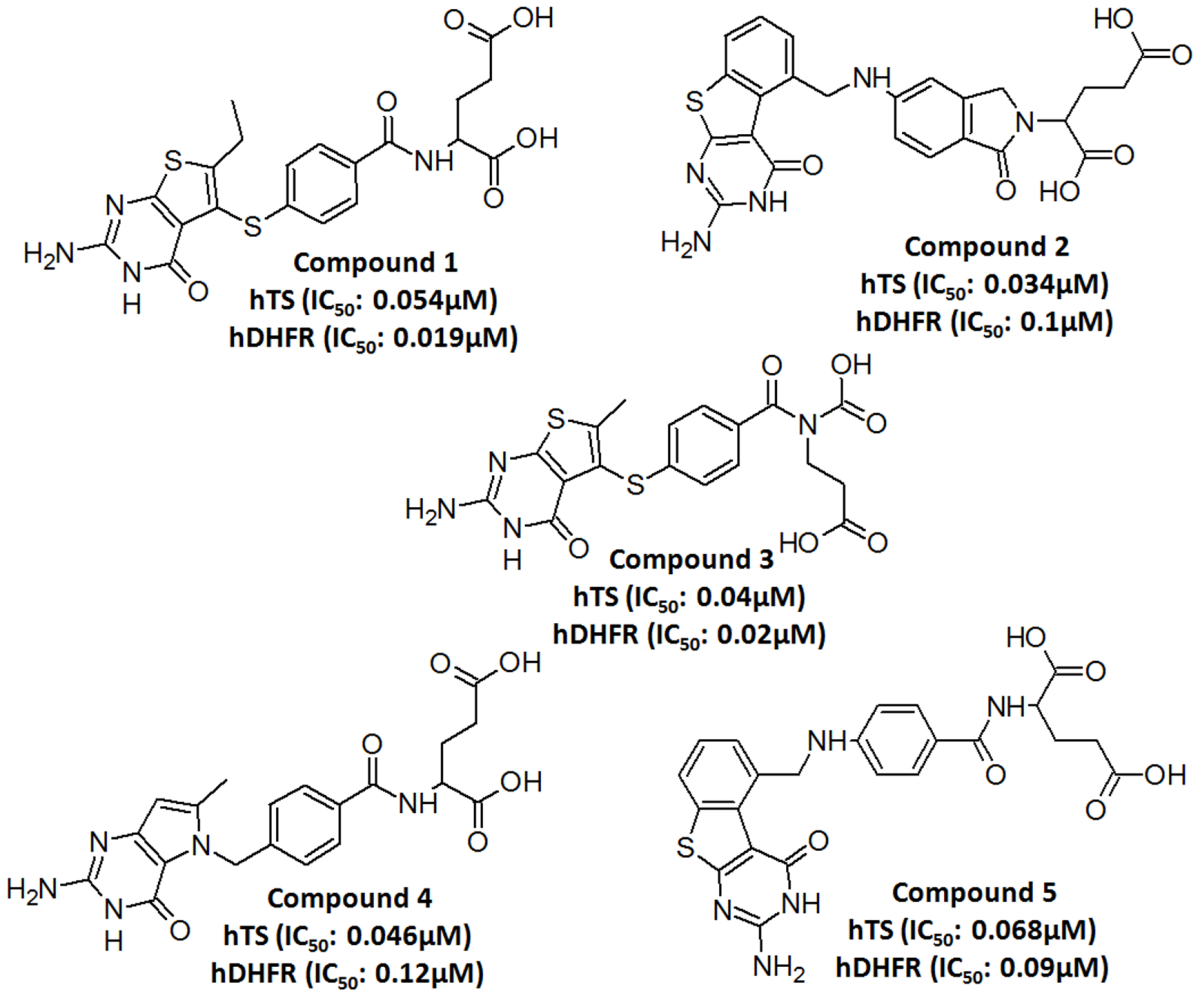
Training set compounds used in common feature pharmacophore generation.

Several scientific resources were searched for the compounds with the experimentally known dual inhibition for hTS and hDHFR. This survey revealed that folate analogue with 2-amino-4-oxo or 2-methyl-4-oxo substitution in the pyrimidine ring is considered important for potent TS inhibitory activity, for example, the clinically used pemetrexed (PMX) and raltiterxed (RTX) [18], [22]. In contrast, folate analogues that inhibit DHFR generally contain 2,4-diamino substitution in the pyrimidine ring for potent DHFR inhibitory activity, typified by methotrexate (MTX), a DHFR inhibitor that has been a mainstay in cancer chemotherapy [26]. Accordingly, five diverse and experimentally known potent dual inhibitors of hTS and hDHFR with these chemical moieties in their structures were selected as training set and were employed in common feature pharmacophore generation calculation (Figure 2) [33]–[36]. Maximum omit feature value of 0 was assigned to all compounds in the training set. Energy minimization process was performed with *CHARMM* forcefield for all the compounds in training set. Poling algorithm was applied to generate a maximum of 255 diverse conformations with the energy threshold of 20 kcal mol^-1^ above the calculated energy minimum for every compound in the dataset. These conformers were generated using *Diverse Conformer Generation* protocol running with *Best/Flexible* conformer generation option as available in DS. All five training set compounds associated with their conformations were used in common feature pharmacophore generation. *HipHop* module of the catalyst which was popularly known for Common Feature Pharmacophore Generation is available in DS as *Common Feature Pharmacophore Generation* protocol. This protocol was used to develop pharmacophore model. *Feature Mapping* protocol was used to identify common features shared by a training set. As predicted, hydrogen bond acceptor (HBA), hydrogen bond donor (HBD) and hydrophobic aromatic (HY_AR) features were selected during the pharmacophore generation. Each hypothesis generation run returns 10 possible pharmacophore hypotheses having a different arrangement of constituent features and sorts them according to the ranking scores. Redundant hypotheses that have the same chemical characteristics and nearly the same distances between these functions were deleted. The hypothesis with diverse configuration was selected as final model according to ranking scores and best fit values. The reliability of the generated pharmacophore model was validated on the basis of presence of chemical features essential to interact with the key amino acids in the active sites of target proteins.

### Ligand Pharmacophore Mapping

The hit compounds obtained from the molecular docking experiments performed both for the hTS and hDHFR with druglike Maybridge database are used in this study along with the pharmacophore model generated for hTS and hDHFR. *Ligand Pharmacophore Mapping* protocol as implemented in DS is used to map all the hit compounds upon the generated pharmacophore model with the *Best Flexible* Search option. The compounds that mapped well on the pharmacophore model were selected as possible hits for dual inhibition and were utilized for their further validation through their binding energy calculations using AutoDock.

### Molecular Docking Study Using Autodock 4.2

Autodock 4.2 was used to calculate the binding energies of the possible hits along with training set compounds at the active sites of both hTS and hDHFR enzymes. As no scoring function employed in currently available docking programs performs better for all macromolecular targets, a combination of scoring functions from various programs (GOLD and AutoDock in this study) may provide significant value in predicting favorable binding conformations. Binding energies of the selected possible dual hits were calculated using AutoDock as a cross-validation to the GOLD predictions. AutoDock consumes more calculation time yet envisages the binding conformations more precisely [37]. It also computes torsional energy which gives rise to the binding energy of the docked compound. The starting proteins for hTS and hDHFR were prepared from their high resolution crystal structures (PDB ID: 1HVY for hTS, PDB ID: 1U72 for hDHFR) deposited in the Protein Data Bank. Possible dual hits along with the training set compounds were docked using the Lamarckian genetic algorithm (LGA) in the “docking active site”, defined through a grid centered on the ligand of the complex structure. Population size of 150, mutation rate of 0.02, and crossover rate of 0.8 were set as the parameters. The default grid spacing (0.375 Å) was used. Simulations were performed using up to 2.5 million energy evaluations with a maximum of 27 000 generations. Each simulation was performed 10 times, yielding 10 docked conformations. The lowest energy conformations were regarded as the binding conformations between ligands and the protein.

### Optimization Studies

One of the final hit compounds was used as lead for further optimization. Various substitutions were made at its side chains. These optimized compounds were also subjected to map pharmacophore model which is generated from experimentally known potent dual inhibitors of hTS and hDHFR enzymes. Moreover, molecular docking experiments using both GOLD and AutoDock programs were also performed for these optimized compounds. Synthetic accessibility scores for all the optimized compounds were used to validate the synthetic possibilities. SYLVIA v 1.0 program from the Molecular Networks group was employed to calculate the synthetic accessibility of these optimized compounds [38], [39]. The estimation of synthetic accessibility using SYLVIA provides a number between 1 and 10 for compounds that are very easy to synthesize and compounds that are very difficult to synthesize, respectively. The method for calculating synthetic accessibility takes account of a variety of criteria such as complexity of the molecular structure, complexity of the ring system, number of stereo centers, similarity to commercially available compounds, and potential for using powerful synthetic reactions. These criteria have been individually weighted to provide a single value for synthetic accessibility.

## Results and Discussion

### Strategy for Designing Dual Inhibitors

Computer-based virtual screening is a quite useful tool in drug design as it is a cost-effective and time saving process. Virtual screening methods can be divided into two broad categories: structure-based, and ligand-based. In case, the three-dimensional (3D) structure of the target receptor or of its binding site is available, docking is a highly effective technique for virtual screening. The pharmacophore model which is an interpretation of the interaction between a receptor and a ligand is also clearly established as one of the successful computational tools in rational drug design. When a set of active ligands is available, it is possible to compute their shared pharmacophore. Keeping this in view, a new strategy integrating structure-based and ligand-based approaches to identify small molecules modulating multiple targets is presented in this study. This strategy merges the structure-based information about the key elements in protein-ligand binding with the dual ligand-based pharmacophore model derived from experimentally known dual inhibitors for design of multitarget drugs.

**Figure 3:**
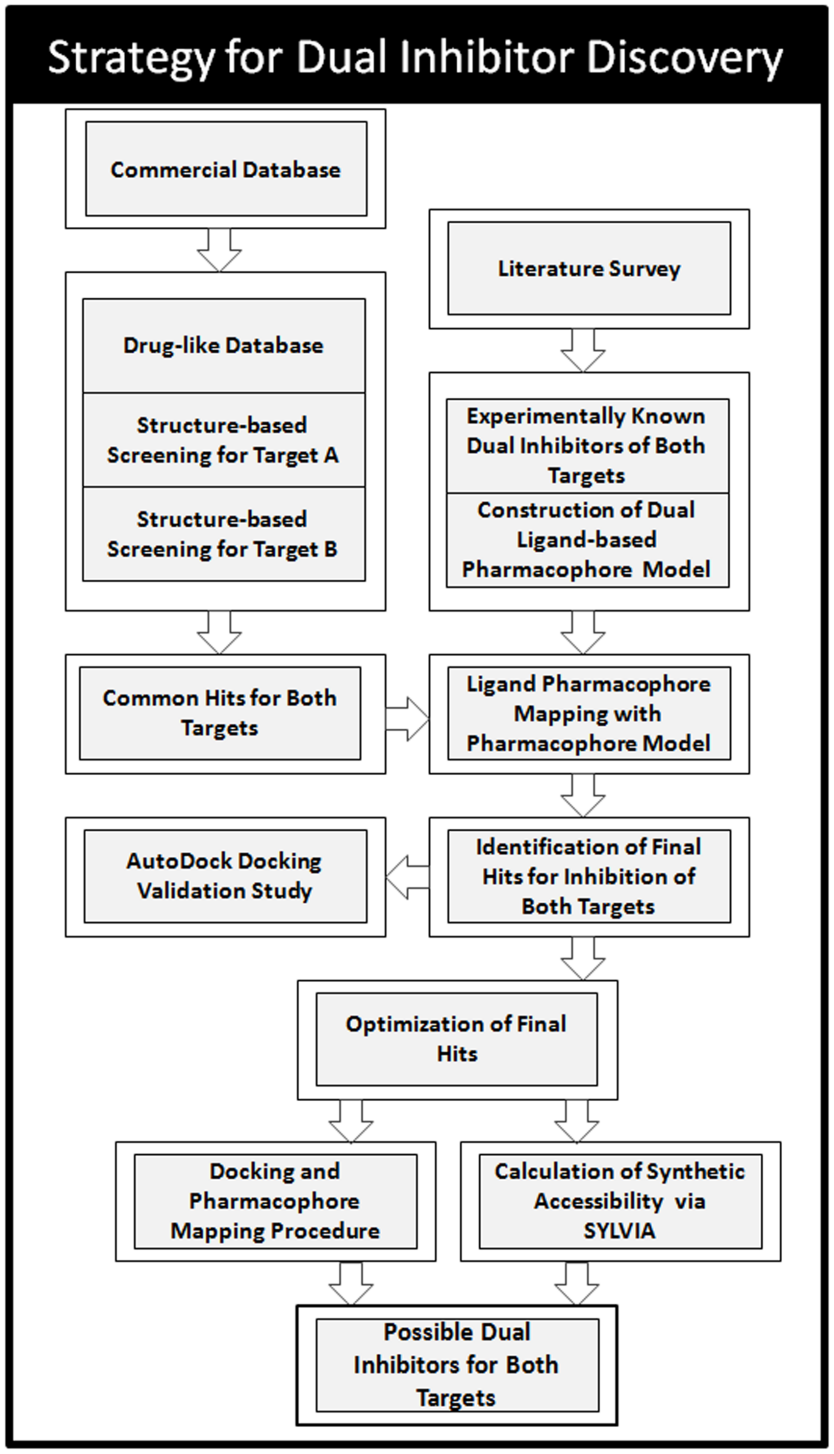
Strategy of dual inhibitor discovery employed in this study.

Our strategy (Figure 3) to discover dual target inhibitors starts from the preparation of a druglike database which is further docked into the active site of one of the target proteins thus predicting the binding conformations and molecular interactions of small molecules of database. On the basis of the binding mode analysis, compounds with good binding characteristics and showing strong interactions with key amino acids at the active site of first protein target were chosen for further processing. In next step, selected compounds were docked into the active site of the second protein target in order to examine their binding affinity for this protein. The binding modes of all the docked compounds were analyzed for their molecular interactions at the active site. The compounds showing strong key interactions at the active site were considered as possible dual hits and were chosen for the next step. After the completion of protein-based virtual screenings, in the third step of dual inhibition process, a common feature pharmacophore model was developed from experimentally known dual inhibitors of both the target proteins. Fourth step involves the mapping of docking hits of both target proteins over the derived pharmacophore model in order to find out, whether these hit compounds possess the very basic chemical features which are present in currently available dual inhibitors of hTS and hDHFR, and whether their binding conformations accommodate the common pharmacophore. Moreover, mapping also helped us to evade the difficulty stemmed from overestimation of the affinity of weak binders producing false positives during docking studies. The compounds scoring the best fit values were docked again to the active site of both the target proteins using another molecular docking program AutoDock to check the binding energies of chosen compounds at the active sites of hTS and hDHFR. Finally, compounds that bind strongly at both the active sites, fit well upon the dual ligand-based pharmacophore model, and exhibit lowest binding energies were regarded as final hits for dual inhibition of hTS and hDHFR. After the selection of final possible dual inhibitors for hTS and hDHFR enzymes, one of the final hits was used as lead for further optimization. The complete strategy followed in this study was also repeated for the optimized compounds to ensure that the optimized hits really fit the generated pharmacophore model and active sites of both the targets.

### Molecular Docking of Druglike Database for Target Proteins

Maybridge database containing 59 652 compounds was used for docking into the active sites of hTS and hDHFR enzymes. Prior to protein-based virtual screening experiments, this database was transformed to druglike databases by *Prepare Ligands* and *ADMET Descriptors* protocols of DS. *Prepare Ligands* protocol eradicated the duplicate structures, fixed bad valencies, and calculated 3D coordinates of all the compounds. *ADMET Descriptors* protocol calculated various properties such as aqueous solubility, blood brain barrier penetration, CYP2D6 binding, hepatotoxicity, intestinal absorption, and plasma protein binding. Calculating ADMET descriptors early in the development of a drug is important to avoid elimination of compounds with unfavorable ADMET characteristics later in the drug development process. Finally, 4966 druglike compounds were selected and were applied subsequently in protein-based virtual screening. The druglike database was docked at the active site of preprocessed protein structure of hDHFRF using GOLD. The experimentally known dual inhibitors of hTS and hDHFR present in the training set were also docked with the same parameters used in database docking. Compound 1 which showed maximum inhibition activity against hDHFR, has scored a GOLD fitness score of 76.448 along with the strong molecular interactions with the key active site residues. The 73 hit compounds from the druglike database scored a GOLD fitness score greater than 70 and were selected for further docking experiment with second target protein hTS. The docking of these 73 possible hDHFR inhibitors along with the training set compounds at the active site of hTS was performed and docking results were evaluated. Compound 2 of training set which showed maximum inhibition activity against hTS, scored GOLD fitness score of 78.220. The scrutiny of bonding conformations of docked hit compounds showed that 32 out of 73 compounds showed GOLD fitness score greater than 70 and also formed strong interactions with active site of enzyme. Thus, these 32 compounds were selected as final leads for further processing in dual inhibition strategy.

### Generation and Selection of a Common Feature Pharmacophore Model for Inhibition of hTS and hDHFR

**Figure 4:**
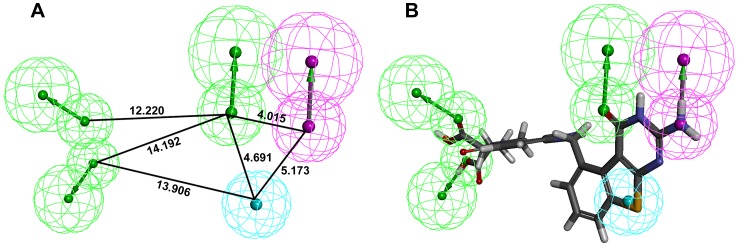
Generated pharmacophore model (Dual_Pharma) along with its interfeature distance (A), and its overlay on compound 1 of the training set (B).

Common feature pharmacophore model was generated for the target proteins using a set of experimentally known dual inhibitors of hTS and hDHFR. With the aim of acquiring a best model, numerous common feature pharmacophore generation runs were performed by altering the parameters such as minimum interfeature distance values, maximum omit feature, and the permutation of pharmacophoric features. The qualitative top ten pharmacophore models were developed (Table 1) using Common *Feature Pharmacophore Generation*/*DS* to identify the common features necessary to inhibit both hTS and hDHFR enzymes. Direct and partial hit mask value of ‘1′ and ‘0′ for models connoted that the compounds present in dataset were well mapped to all the chemical features in the models and there is no partial mapping or missing features. The *Cluster analysis* was used to evaluate and categorize the difference between the compositions of models’ chemical features and locations. These models could be roughly classified into two clusters according to the pharmacophoric features presented. The models in cluster I identified five functional features, including three HBA, one HBD, and one HY_AR feature. The models in cluster II also recognized five functional features, with three HBA, one HBD, and one ring aromatic (RA). A closer scrutiny of the pharmacophore models in each cluster revealed subtle discrepancy among models. The distances between some pharmacophoric features in all models were rather constant, whereas some distances fluctuated in a relatively broad range, which indicated divergent tolerance of different features to spatial variation and provided rationale for further structural modification and optimization. The top two models in cluster I showed comparatively higher ranking score and best fit values of the training set compounds, therefore, these two models were further evaluated to find the best model. These pharmacophore models have scored same ranking score; therefore, an analysis of the best fit values of the training set compounds was carried out to choose the best model. The calculated best fit values designated Model 1 as the best and final dual ligand-based pharmacophore model (Dual_Pharma) (Figure 4A). This final Dual_Pharma which consists of three HBA, one HBD, and one HY_AR feature was further overlaid on the most active compound of training set (Figure 4B). The most active compound 1 could map all the features of the Dual_Pharma, with a fit value of 4.99.

**Table 1:** Summary of the Dual Pharmacophore Models Generated for hTS and hDHFR.

hypothesis	features	rank	directhit	partialhit	max.fit
Hypo1	ZDAAA	88.594	11111	00000	5
Hypo2	ZDAAA	88.594	11111	00000	5
Hypo3	ZDAAA	88.438	11111	00000	5
Hypo4	RDAAA	88.257	11111	00000	5
Hypo5	RDAAA	88.257	11111	00000	5
Hypo6	RDAAA	88.257	11111	00000	5
Hypo7	RDAAA	88.257	11111	00000	5
Hypo8	ZDAAA	88.196	11111	00000	5
Hypo9	ZDAAA	88.196	11111	00000	5
Hypo10	ZDAAA	87.933	11111	00000	5

### Comparison with the Models of Literature

Our dual ligand-based pharmacophore model is in agreement with the information provided by the former studies. The importance of HBA and HBD is confirmed for DHFR and TS inhibition. Moreover, presence of hydrophobic aromatic features is also considered significant for inhibition of DHFR and TS enzymes [40]–[42].

### Validation of Common Feature Pharmacophore Model

**Figure 5:**
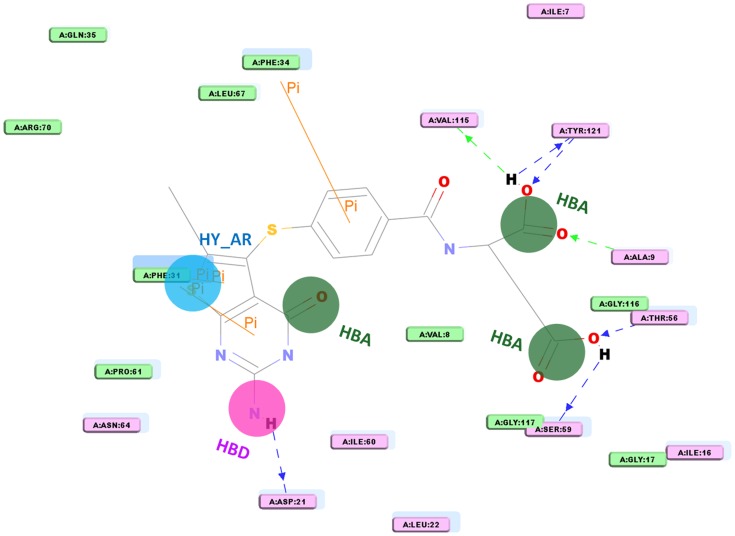
Ligand-protein interaction diagram of hDHFR-inhibitor complex (compound 1 in the training set). The pharmacophore mapping of the same compound is also illustrated. HBA, hydrogen bond acceptor; HBD, hydrogen bond donor; HY_AR, hydrophobic aromatic. The locations of amino acid residues are represented in rectangular boxes, where pink and green colors denote both the hydrogen bond acceptor/donor and nonpolar contacts, respectively.

**Figure 6:**
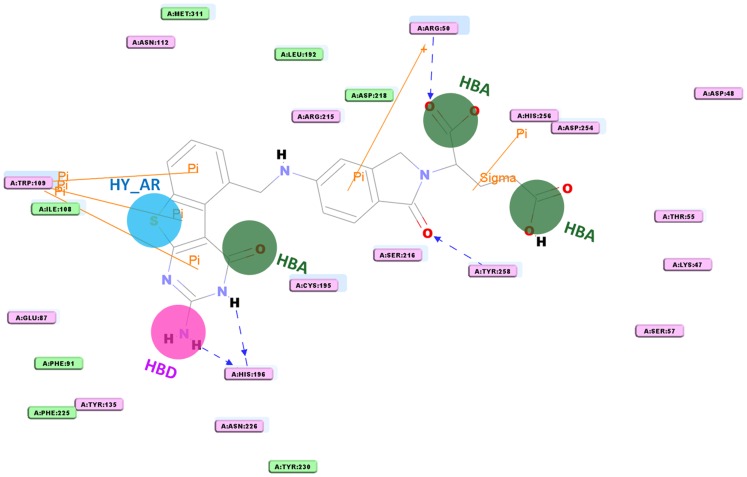
Ligand-protein interaction diagram of hTS-inhibitor complex (compound 2 in the training set). The pharmacophore mapping of the same compound is also illustrated. HBA, hydrogen bond acceptor; HBD, hydrogen bond donor; HY_AR, hydrophobic aromatic. The locations of amino acid residues are represented in rectangular boxes, where pink and green colors denote both the hydrogen bond acceptor/donor and nonpolar contacts, respectively.

The purpose of the pharmacophore validation is to appraise the quality of a pharmacophore model. The reliability of the Dual_Pharma developed from the dual inhibitors of hTS and hDHFR was validated on basis of the existence of chemical features crucial to interact with the important amino acids in the active sites of both target proteins. To find out the existence of chemical features that are complementary to the active site of hDHFR enzyme, one of the training set compounds was overlaid on Dual_Pharma and its binding conformation was generated from the molecular docking process. Furthermore, ligand interaction diagram was generated for the hDHFR-inhibitor complex by using DS which illustrated the amino acids complemented to every feature present in the pharmacophore model (Figure 5). Dual_Pharma consists of three HBA, one HBD, and one HY_AR feature. Overlay of the bound inhibitor on Dual_Pharma connoted that chemical features of pharmacophore model were located in such a way that HY_AR feature was found to make important hydrophobic contact with Phe31 which is considered an essential amino acid for DHFR inhibition [33].Moreover, orientation of HBD feature in the binding site made it possible that it could interact with essential residues like Ile60, Asp21, and Leu22. While, all three HBA features were positioned in such a manner that instigated interactions with residues like Val115, Tyr121, Ala9, and Thr56 and others at the active site region of hDHFR. In case of hTS enzyme, ligand interaction diagram was also generated for the hTS-inhibitor complex which manifested that HBA features were able to form imperative interactions with key residues like Asp218, Arg50, Tyr258, and Ser216 (Figure 6). The HY_AR and HBD features also showed important contacts with Trp109, Ile108, and His196 [43].Thus, these annotations associated with the features present in the developed pharmacophore model compared with the interaction spots at the active site regions of hTS and hDHFR have denoted the importance of the generated dual pharmacophore for having the very basic chemical features necessary for inhibition of both target proteins.

### Ligand Pharmacophore Mapping and Identification of Final Dual Hits

The hit compounds obtained from the molecular docking experiments of hTS and hDHFR are employed in this procedure along with the pharmacophore model developed from dual inhibitors of hTS and hDHFR. In order to find out the presence of features which are imperative for strong enzyme-ligand binding interactions for both enzymes in the hit compounds, the binding conformations of the 32 hit compounds which scored GOLD fitness score over 70 for each target protein were mapped over the validated Dual_Pharma using *Ligand Pharmacophore Mapping* protocol of DS. Out of 32 hits, three structurally diverse hit compounds that mapped well on the developed pharmacophore model, showed key interactions with the active site residues, and also scored highest GOLD docking score for both target enzymes were selected as final hits for dual inhibition of hTS and hDHFR (Table 2).

**Table 2:** GOLD Fitness Scores and AutoDock Binding Energies of Final Possible Dual Inhibitors of hTS and hDHFR.

	hTS Binding		hDHFR Binding	
Compound	GOLD Fitness Score	Binding Energy (kcal/mol)	GOLD Fitness Score	Binding Energy (kcal/mol)
ML00100	83.743	−6.32	78.506	−6.77
SPB07954	81.509	−5.78	76.042	−6.02
HTS07361	82.372	−6.17	72.698	−7.03

In order to further validate the performance of docking procedure, database screening process was performed using common feature pharmacophore model labeled as Dual_Pharma. Overall, 14 compounds of druglike Maybridge database mapped the features of Dual_Pharma. Three final hits were also among these 14 compounds retrieved by Dual_Pharma through database screening process. However, when GOLD docking score of newly retrieved 11 compounds was checked, it showed much less score for both targets as compared to final three hits. Thus, the result of this database screening process further validated the good performance of docking procedure in identifying potentially potent dual inhibitors of hTS and hDHFR.

**Figure 7:**
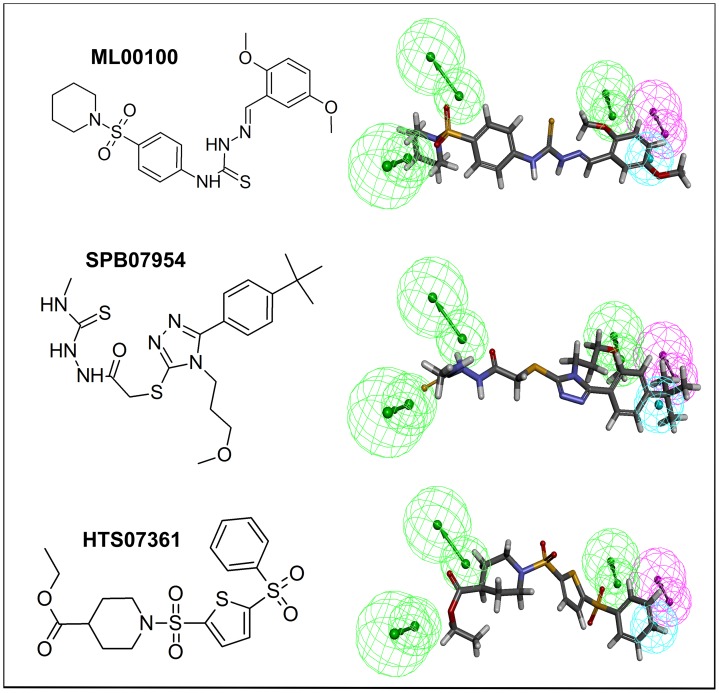
Chemical structures of identified hits for dual inhibition and their overlay on pharmacophore model (Dual_Pharma).

The 2D structures of final three hits ML00100, SPB07954, and HTS07361 along with their mapping over Dual_Pharma are shown in Figure 7. These final dual hits were utilized for their further validation as potential dual inhibitors through computation of their binding affinity for the target proteins via another molecular docking study using AutoDock.

### Molecular Docking Study Using Autodock 4.2

The binding energies of the final hits along with training set compounds were calculated at the active sites of both hTS and hDHFR enzymes as a cross-validation to the GOLD predictions. AutoDock 4.2 is based on a Lamarckian genetic algorithm (LGA) method. Basically, this program determines total interaction energies between random pairs of ligands and various selected portions of protein to determine docking poses. Final three hits along with the training set compounds were docked using LGA in the “docking active site”, defined through a grid for hTS and hDHFR. Although, AutoDock consumes more calculation time yet envisages the binding conformations more precisely. In case of hTS enzyme, Autodock docking results showed that all the three final hits have gained better binding energy values compared to the binding energies of experimentally known potent dual inhibitors of the training set. Moreover, Autodock docking of the final hit compounds for hDHFR also showed better or similar binding energies compared to the training set compounds. Thus, upshots of Autodock docking study not only validate the GOLD predictions but also envisage the three final hits for possible dual inhibition of TS and DHFR.

### Binding Modes of the Identified Hits

**Figure 8:**
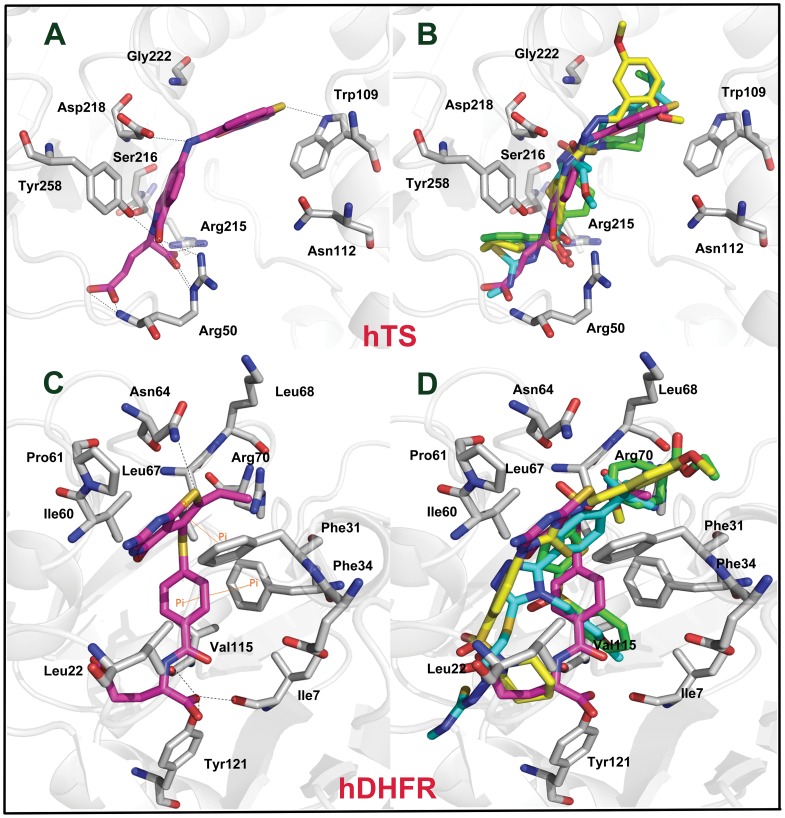
Binding modes of (A) compound 2 from the training set (B) overlay of all three dual hit compounds in the active site of hTS. (**C**) compound 1 from the training set, and (**D**) overlay of all three dual hit compounds in the active site of hDHFR. Key protein residues and ligands are represented by thick sticks. Hydrogen atoms have been removed for clarity.

Compound 1 and compound 2 of the training set which showed potent activity for hDHFR and hTS, respectively, were analyzed for their binding modes and the molecular interactions. Compound 1 was considered as reference to evaluate the binding modes of the final dual hits at the active site of hDHFR. This compound has formed hydrogen bond interactions with key residues like Ile7, Val115, Asn64, and Tyr121 (Figure 8A). Moreover, very important π^…^π interactions between compound 1 and key residues Phe31and Phe34 were also observed. Compound 2 has shown interactions at the active site of hTS with key residues including Arg50, Trp109, Asp218, and Tyr258. This compound was used as standard compound for binding mode analysis of final hits at hTS active site (Figure 8C). Molecular overlay of final three hits at the active sites of hTS and hDHFR with compounds 1 and 2 is depicted in Figure 8. As, several crystal structures of hDHFR and hTS enzymes complexed with various inhibitors are presented in the literature, therefore, molecular interactions between the target protein systems and the diverse inhibitors were also scrutinized from the ligplot figures accessible through the PDBSum database for further validation of the binding modes of final dual hit compounds.

### Binding Mode of ML00100

**Figure 9:**
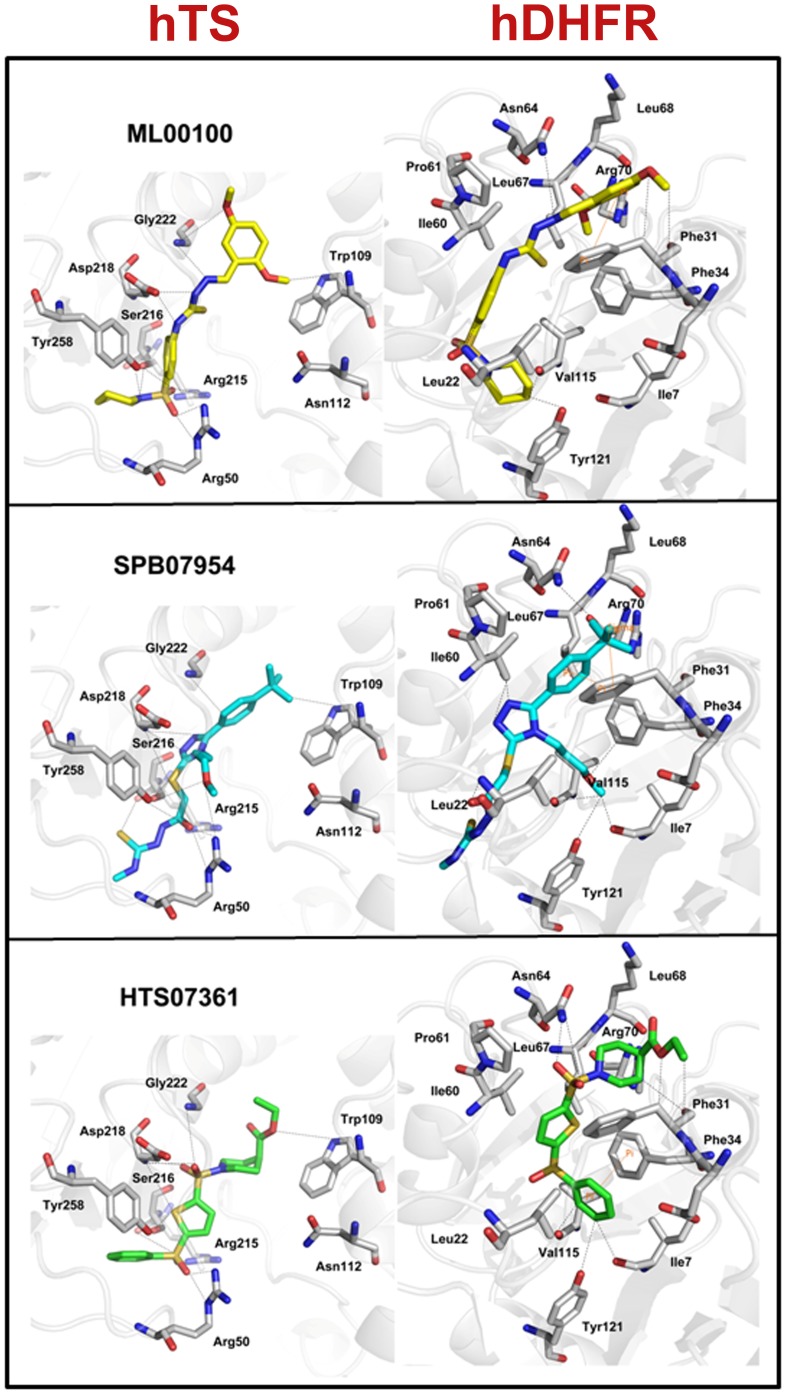
Binding modes of all three dual hit compounds in the active sites of hTS and hDHFR. Key protein residues and hit compounds are represented by thick sticks. Hydrogen atoms have been removed for clarity.

The binding modes of ML00100 within the active sites of hTS and hDHFR have been elucidated. For hTS, this compound has scored a GOLD fitness score of 83.743 and has demonstrated interactions with the key residues like Trp109, Arg50, Asp215, Ser216, Asp218, Gly222, and Tyr258 (Figure 9). The meticulous analysis of binding conformation of ML00100 in the binding pocket of hTS revealed that1,4-dimethoxybenzene ring is making important hydrophobic contact with Trp109. Previous study has also indicated that this kind of interaction with Trp109 enhances the potent inhibitory activity against hTS [35]. Furthermore, various close contacts between the oxygen atoms of sulfonyl group and Arg50 (2.778 Å, 3.152 Å) were observed. The nitrogen atoms of ML00100 compound also formed hydrogen bonds with the important residues like Arg 215, Asp218 (2.169 Å, 2.644 Å), Gly222 (3.389 Å), and Tyr258 (3.668 Å). For hDHFR, this compound has scored a GOLD fitness score of 78.506 and has shown interactions with the important residues including Leu22, Phe31, Asn64, Val115, and Tyr121. The 1,4-dimethoxybenzene ring of the ML00100 has established imperative π^…^π interactions with Phe31 in the binding pocket of hDHFR. This key interaction has significant role in driving the hit compound to adopt an appropriate bioactive conformation oriented in the active site of enzyme. Moreover, the 1,4-dimethoxybenzene ring oxygen and the nitrogen atom bonded with this ring system made hydrogen bond contacts with Phe31 and amide of Asn64. The pyridine group of ML00100 has also formed hydrophobic interactions with the carbonyl of Val115 (3.897 Å), and hydroxyl of Tyr121 (3.732 Å). On the whole, this compound has gained significant hydrophobic interactions at both the active sites.

**Figure 10:**
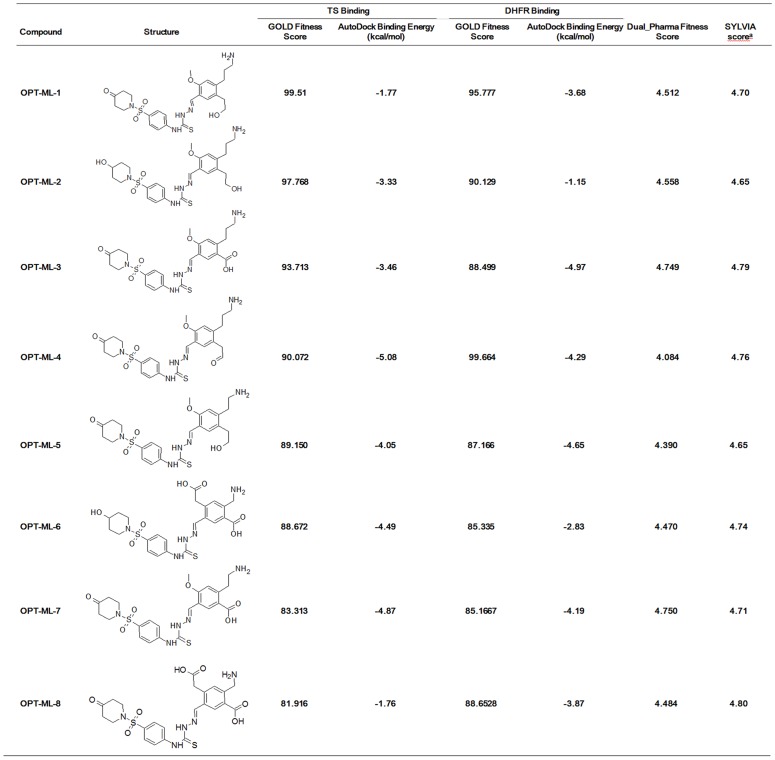
GOLD fitness scores, AutoDock binding energies, and SYLVIA synthetic accessibility scores of top 8 optimized hit compounds.

### Binding Mode of SPB07954

This compound has scored the GOLD fitness scores of 81.509 for hTS binding and 76.042 for hDHFR binding. Binding at the active site of hTS revealed that considerable interactions are formed between SPB07954 and important residues Trp109, Arg50, Asp215, Ser216, Asp218, Gly222, and Tyr258 (Figure 9). A vital hydrophobic interaction was formed between the tert-butylbenzene ring system of SPB07954 and indole moiety of Trp109. Moreover, nitrogen atoms of triazole ring exhibited hydrogen bond interactions with Asp218 (2.321 Å, 2.627 Å), and Ser216 (3.201 Å) residues. The central sulfur atom of the SPB07954 compound was also involved in interactions with the hydroxyl group of Tyr258 (3.143 Å), and Ser216 (2.520 Å). On the whole, numerous key interactions were observed between SPB07954 and binding pocket residues of hTS. In terms of hDHFR, SPB07954 illustrated various kind of interactions such as π^…^π, π^…^σ, and hydrogen bonding interactions with the important residues like Ile7, Phe31, Ile60, Asn64, Val115, and Tyr121. The tert-butylbenzene ring of SPB07954 is involved in crucial π^…^π, π^…^σ interactions with the phenyl ring of key residue Phe31. These interactions instigated the SPB07954 compound to gain a proper bioactive conformation positioned in the binding pocket of enzyme. The central triazole ring system formed hydrogen bonding with the Ile60 (3.411 Å, 3.193 Å). Whereas, one of the carbonyl group formed hydrogen bond contact with amide of Leu22. Moreover, methoxypropane moiety formed various hydrophobic contacts with the backbone of Ile7 (2.818 Å), Val115 (3.282 Å), and Tyr121 (3.506 Å).

### Binding Mode of HTS07361

HTS07361was predicted with top GOLD fitness scores of 82.371 and 72.698 for hTS and hDHFR, respectively. In case of hTS binding, this compound revealed a network of interactions with important residues like Arg50, Trp109, Ser216, Asp218, Gly222, and Tyr258 (Figure 9). The oxygen atom of piperidine-4-carboxylate ring system formed key hydrophobic interaction with the indole moiety of Trp109. Both the oxygen atoms of the sulfonyl group anchored with piperidine-4-carboxylate ring of HTS07361 compound are engaged in short-ranged hydrogen bond interactions with Asp218 (1.731 Å, 3.256 Å), and Gly222 (3.342 Å). While, sulfur atom of thiophene ring and the oxygen atoms of another sulfonyl group of HTS07361 have also formed numerous strong hydrogen bonding interactions with amide groups of Arg50 (2.174 Å, 3.073 Å) and Arg215 (1.563 Å, 2.921 Å), hydroxyl of Ser216 (2.147 Å), amide of Asp218 (3.768 Å), and hydroxyl of Tyr258 (3.737 Å). The elucidation of binding mode of HTS07361 for hDHFR exhibited several interactions with the active site residues like Ile7, Phe31, Phe34, Asn64, Val115, and Tyr121.The imperative π^…^π interactions between the phenyl rings of HTS07361 and Phe34 were also observed. This aromatic stacking seems to play a decisive role for proper orientation of HTS07361 in the binding pocket. The same phenyl group of this hit compound is also embroiled in close hydrogen bonding to the CO of Ile7 (2.615 Å), Val115 (2.169 Å), and hydroxyl of Tyr121 (3.056 Å). Moreover, the oxygen atoms of the sulfonyl group affixed with piperidine-4-carboxylate ring of HTS07361 compound have also formed significant hydrophobic interactions with Phe31, and Asn64 in the active site region.

In summary, the interactions exhibited by the three dual hits are analogous to the key interactions of experimentally known dual inhibitors of hTS and hDHFR at the active site regions of the target enzymes [33]–[36], [44].

### Optimization Studies

The top dual hit ML00100 of hTS and hDHFR was subjected for further optimization studies. Diverse substitutions were made in its structure with the aim of improving its binding affinity towards the catalytically active amino acids. The optimized hits were docked into the active sites of hTS and hDHFR using GOLD program with the same parameters used to dock the direct database hits. The binding modes of optimized hit compounds were analyzed for molecular interactions with the essential amino acids like Trp109, Arg50, Asp215, Ser216, Asp218, Gly222, and Tyr258 at hTS binding site. In terms of hDHFR, binding mode analysis was made to ensure that optimized hits hold imperative interactions with key residues like Ile7, Leu22, Phe31, Phe34, Ile60, pro61, Asn64, Val115, and Tyr121. Furthermore, the binding energies of the optimized hits at the active sites of both hTS and hDHFR enzymes were also calculated using Autodock. These optimized compounds were further mapped to Dual_Pharma generated from experimentally known potent dual inhibitors of hTS and hDHFR enzymes. Finally, top eight optimized hits which showed maximum GOLD fitness scores and lowest binding energies for both targets along with high fit value for Dual_Pharma were also selected as final hits for dual inhibition of hTS and hDHFR along with direct database hits (Figure 10). As last step of our dual inhibition strategy, the synthetic accessibility of the final 8 optimized hits was measured using SYLVIA 1.0 program. The SYLVIA score for most of the final optimized hits illustrates that these compounds are easy to be synthesized.

### Conclusion

We developed a novel computational approach by integrating the affinity predictions from structure-based virtual screening with dual ligand-based common feature pharmacophore to discover potential dual inhibitors of hTS and hDHFR as antitumor agents. As first step, a druglike database was prepared and was utilized to perform dual-target docking studies. From results of docking studies, compounds which formed strong interactions at the active site for both target proteins were identified and selected for further evaluation. Furthermore, a common feature pharmacophore model was developed from experimentally known dual inhibitors of hTS and hDHFR. This pharmacophore was mapped over the compounds which were identified through dual-target docking studies. The pharmacophore mapping procedure not only facilitated us in eliminating the compounds which do not possess basic chemical features necessary for dual inhibition of target enzymes, but, also helped us to evade the difficulty stemmed from overestimation of the affinity of weak binders producing false positives during docking studies. Moreover, binding energies of the selected possible dual hits were also calculated using AutoDock as a cross-validation to the GOLD predictions. Finally, three structurally diverse hit compounds that showed key interactions at both the active sites, fit well upon the dual ligand-based pharmacophore model, and exhibit lowest binding energies were regarded as final hits for dual inhibition of hTS and hDHFR. In addition, optimization studies were performed for a final dual hit compound and eight optimized dual hits demonstrating excellent binding features at target protein systems were developed. Synthetic accessibility of developed optimized hits was also computed using SYLVIA. The possible dual inhibitors along with optimized hits may find clinical utility as antitumor, antimicrobial, and antiprotozoal agents. On the whole, the strategy used in the current study could be a promising computational approach and may be generally applicable to other dual target drug designs.
